# Fisher–Shannon Investigation of the Effect of Nonlinearity of Discrete Langevin Model on Behavior of Extremes in Generated Time Series

**DOI:** 10.3390/e25121650

**Published:** 2023-12-12

**Authors:** Luciano Telesca, Zbigniew Czechowski

**Affiliations:** 1 Institute of Methodologies for Environmental Analysis, National Research Council, 85050 Tito, Italy; 2 Institute of Geophysics, Polish Academy of Sciences, 01-452 Warsaw, Poland; zczech@igf.edu.pl

**Keywords:** nonlinear Langevin equation, time series, Fisher–Shannon plane, run theory, extremes

## Abstract

Diverse forms of nonlinearity within stochastic equations give rise to varying dynamics in processes, which may influence the behavior of extreme values. This study focuses on two nonlinear models of the discrete Langevin equation: one with a fixed diffusion function (M1) and the other with a fixed marginal distribution (M2), both characterized by a nonlinearity parameter. Extremes are defined according to the run theory with thresholds based on percentiles. The behavior of inter-extreme times and run lengths is examined by employing Fisher’s Information Measure and the Shannon Entropy. Our findings reveal a clear relationship between the entropic and informational measures and the nonlinearity of model M1—these measures decrease as the nonlinearity parameter increases. Similar relationships are evident for the M2 model, albeit to a lesser extent, even though the background data’s marginal distribution remains unaffected by this parameter. As thresholds increase, both the values of Fisher’s Information Measure and the Shannon Entropy also increase.

## 1. Introduction

Numerous complex natural phenomena, observed via time series, are modeled using stochastic processes due to limited knowledge about the underlying intrinsic processes. Many of these phenomena are nonlinear in nature, leading to the prevalent use of nonlinear stochastic models to describe them accurately.

The Langevin equation stands as a widely employed nonlinear stochastic model for time series. It is used to describe the evolution of a wide class of stochastic diffusion Markov processes and is associated with the equivalent Fokker–Planck equation for the time evolution of the distribution function. One of its significant advantages is its capability to model a broad spectrum of distributions, ranging from Gaussian to those with long tails. The equation was applied in many fields of science, physics, chemistry, biology, and finance (see Allen [1]), being a handy nonlinear model of time series. Its reconstruction from the data has been extensively developed (for details, refer to the comprehensive review paper by Friedrich et al. [2]) and applied to various natural time series, like rough surfaces, porous media, physiology, financial data, turbulence, geophysical processes, and others (see [2,3,4,5]).

Different types of nonlinearity give rise to varied dynamics within the process, likely influencing the behavior of extremes. However, this specific aspect has not undergone comprehensive investigation to date. Our study aims to illustrate, among other findings, that despite the marginal distribution of the underlying time series (realization of the process) remaining constant against changes in the model’s nonlinearity, the process’ dynamics significantly impact the behavior of extremes.

In this paper, we adopt the definition of extremes following the run theory [6,7,8]. We intend to examine the behavior of inter-extreme times and run lengths by using two informational quantities, Fisher’s Information Measure and the Shannon Entropy. Initially formulated within the context of information theory, these two quantities have found successful application in the analysis of complex dynamics of non-stationary time series [9,10,11,12], particularly in examining dynamical changes in biophysical signals [13,14,15], in atomic systems [16,17], in particulate matter concentration data [18], and in natural processes, like earthquakes [19,20,21] or volcanic eruptions [22]. The Fisher–Shannon method applied to the geoelectrical data enabled the identification of long-term deformation processes in the Taiwan orogeny [23]. Moreover, they were utilized to elucidate laboratory and theoretical earthquake rupture models [24].

## 2. Models

For our analysis, we require a stationary model characterized by a single parameter influencing nonlinearity. This choice facilitates the identification of the desired relationships. Alterations in nonlinearity within the drift and diffusion terms can impact the marginal distribution, potentially extending the tail of the distribution. Alternatively, such changes can be chosen to maintain the invariant form of the marginal distribution. It is reasonable to expect that an increase in the tail within the distribution would impact the behavior of extreme values, given the clear association between long tails and extremes. Nevertheless, a comprehensive and systematic study of these relationships is currently lacking. However, the impact of changes in the process dynamics caused by changes in the nonlinearity of the drift and diffusion terms, while maintaining the same marginal distribution, has not been taken into account and analyzed so far. All the above conditions are met via the Langevin equation model derived in [25], which has an additional important advantage of a simple analytical form of the marginal distribution function with an explicit dependence on the nonlinearity parameter. We employ the discrete nonlinear Langevin equation as a generator for time series, specifically utilizing the forward Euler discrete approximation [26] expressed as:

(1) $$y\left(t+\Delta t\right)=y\left(t\right)+a\left(y\left(t\right)\right)\Delta t+\sqrt{b(y(t))\Delta t}\xi_{t}$$

Here, y(t) represents a stochastic process, Δt is the time step, and $\xi_{t}$ stands for an independent random variable with a normal density. The drift function a(y) and diffusion function b(y) can take the form of either linear or nonlinear functions. In our investigation, we consider two distinct forms of nonlinearity (models M1 and M2), each delineated by a singular parameter. However, a common model exists for both of these classes, and both are particular cases of the model introduced in [25].

For model M1, the diffusion function is defined as b(y)=y, while the drift function a(y) depends on a nonlinearity parameter α, as follows:

(2) $$a\left(y\right)=\frac{1}{2}\left(\frac{1}{y}-y^{\alpha }\right)$$

**Figure 1:**
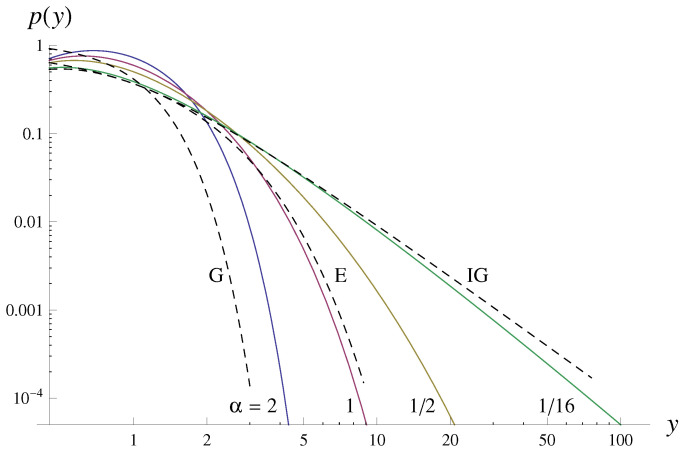
Marginal distribution functions p(y) (see Equation (3)) for α=2,1,1/2,1/16 (log–log plot). For a comparison, three typical distributions are also presented: half-Gaussian (G), exponential (E), and inverse-gamma (IG).

We will explore instances with various α values such as α=2,1,1/2,1/4,1/8,1/16, with y>0. Consequently, the marginal distribution function p(y) takes the following continuous form:

(3) $$p\left(y\right)=\frac{c}{y}e^{-\frac{1}{y}-\frac{1}{\alpha }y^{\alpha }}$$

where *c* is a normalization factor. Function p(y) is the analytical stationary solution of the Fokker–Planck equation, which is associated with the Langevin Equation (1) (see e.g., Equations (12) and (13) in [25]). As the parameter α decreases, the tail of the distribution increases. Figure 1 illustrates the marginal distribution function for half-Gaussian (G) $\frac{2}{\sqrt{\pi }}e^{-y^{2}}$, exponential (E) $e^{-y}$, and inverse-gamma (IG) $\frac{1}{y^{2}}e^{-\left(\frac{1}{y}\right)}$ for comparative purposes.

In model M2, both functions b(y) and a(y) are dependent on the nonlinearity parameter β, i.e.,

(4) $$b\left(y\right)=y^{\beta -1},\;a\left(y\right)=\frac{1}{2}\left(y^{\beta -3}+\left(\beta -2\right)y^{\beta -2}-y^{\beta }\right)$$

but the marginal distribution function has the invariant form

(5) $$p\left(y\right)=\frac{c}{y}e^{-\frac{1}{y}-\frac{1}{2}y^{2}}.$$

This setup allows us to eliminate the dependence of extreme value behaviors from the distribution function. Notably, the form of p(y) mirrors that in Equation (3) for α=2 (i.e., a(y) and b(y) with α=2 in M1 equals a(y) and b(y) with β=2 in M2). We will explore instances with β=2.0,2.2,2.4,2.6,2.8,3.0.

For each α (and β) case, we have generated 10 time series of length n=1,000,000 with a time step dt=0.01 using both models M1 and M2, intending to investigate the behavior of extreme values within these data.

## 3. Definition of Extremes

In this study, the run theory (or crossing theory) [6,7,8] is chosen to define extremes. According to this approach, a run is defined as a sequence of *R* contiguous values above a predetermined threshold, and an extreme can be characterized by a three-component vector (t,m,R), where *t* denotes the time when the run initiates, *m* signifies the magnitude (such as the maximum within the run), and *R* represents the run’s length or run’s duration. The thresholds are defined based on percentiles [27] and are established as a percentile of the distribution of the series’ values. The run theory proves particularly beneficial in estimating the duration of runs above a specific level, such as the time duration of droughts or floods, stock exchange runs, and similar scenarios.

In the cases examined within this study, we assume thresholds above which the extremes are detected to be the 90th, 92.5th, 95th, and 97.5th percentiles of the distribution of the background series’ values. Percentile-based extremes are frequently employed in the analysis of hydrological or climate extremes [27,28,29].

Two quantities can effectively represent a sequence of extremes: (1) The series of inter-extreme times, which denotes the intervals between contiguous extremes. (2) The series of run lengths, highlighting the duration of each extreme event.

## 4. The Fisher–Shannon Information Plane

The Fisher-Shannon Information Plane (FSIP), initially proposed by Vignat and Bercher [11] and then subsequently utilized in several works [30,31,32], is a planar representation where the horizontal and vertical axes are functionals derived from the relevant probability distribution function (PDF), the Shannon Entropy *S*, and Fisher’s Information Measure the FIM, respectively. This tool serves as a convenient means to depict both global and local aspects of the PDFs associated with the studied system on the same information plane.

For a continuous probability distribution function (PDF) f(x) with x∈Δ⊂R and $\int_{\Delta }f\left(x\right)\;dx=1$, its Shannon Entropy is defined as [33]

(6) $$S\left[f\right]=-\int_{\Delta }f\ln f\;dx,$$

a measure of a ‘global’ nature that is not excessively affected by significant changes occurring in localized areas of the distribution’s support set Δ.

This is not true for Fisher’s Information Measure (FIM) that quantifies the gradient of the PDF, being particularly sensitive even to small localized perturbations [34,35,36]. It is defined as

(7) $$F\left[f\right]=\int_{\Delta }\frac{1}{f(x)}{\left(\frac{df(x)}{dx}\right)}^{2}dx.$$

Fisher’s Information Measure (FIM) has diverse interpretations, serving as a gauge for the precision of parameter estimation, the extent of information retrievable from a set of measurements, and a metric indicating the degree of disorder within a system or phenomenon [36]. In the earlier definition of FIM (Equation (7)), the division by f(x) poses difficulties when f(x) becomes too small to be accurately computed. In this case, utilizing probability amplitudes $\psi =\sqrt{f}$, such an issue could be avoided [36,37]. The influence of the gradient operator significantly impacts the contribution of subtle local variations to the value of FIM. Hence, this quantifier is acknowledged as a ‘local’ metric [36].

Considering discrete probability distributions ($P=\left\{p_{j}:j=1,\cdots ,M\right\}$), its Shannon Entropy S[P] [33] is defined as:

(8) $$S\left[P\right]=-\sum_{j=1}^{M}p_{j}\cdot \ln\left(p_{j}\right)$$

When S[P]=0, there is certainty about which of the possible outcomes *j* with probabilities $p_{j}$ will occur, signifying maximal knowledge of the underlying process described by the probability distribution. Conversely, for a uniform distribution, this knowledge is minimal. For a given distribution *P*, the “normalized Shannon entropy” is calculated as:

(9) $$S_{n}\left[P\right]=\frac{S[P]}{S_{\max}}$$

where $S_{\max}=\ln\left(M\right)$.

For the same probability distribution *P*, starting from the expression in terms of real probability amplitudes (Equation (7)), the FIM is defined as:

(10) $$F\left[P\right]=F_{0}\sum_{i=1}^{M-1}{\left[{\left(p_{i+1}\right)}^{\frac{1}{2}}-{\left(p_{i}\right)}^{\frac{1}{2}}\right]}^{2}$$

The normalization constant (see also Supplementary Materials) is defined as:

$$F_{0}=\left\{\begin{array}{cc} 1 & \mathrm{if}\;p_{i^{*}}=1\;\mathrm{for}\;i^{*}=1\;\mathrm{or}\;i^{*}=M\;\mathrm{and}\;p_{i}=0\;\forall \;i\neq i^{*}\\ \frac{1}{2} & \mathrm{otherwise} \end{array}\right.$$

It has been extensively discussed that this discretization is the best behaved in a discrete environment [38].

In the discrete case, the axes of FSIP are the normalized Shannon Entropy $S_{n}$ and FIM *F*, which are well suited for investigating both global and local features of the probability distribution under study.

## 5. Results

**Figure 2:**
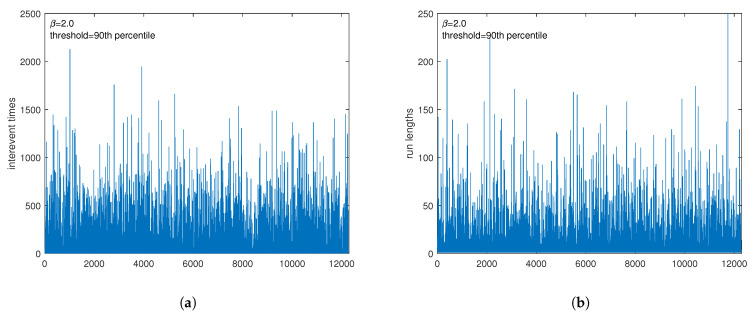
Example of extremes for a time series generated with β=2.0 and threshold = 90th percentile of the distribution of the series’ values: (**a**) interevent times; (**b**) run lengths.

**Figure 3:**
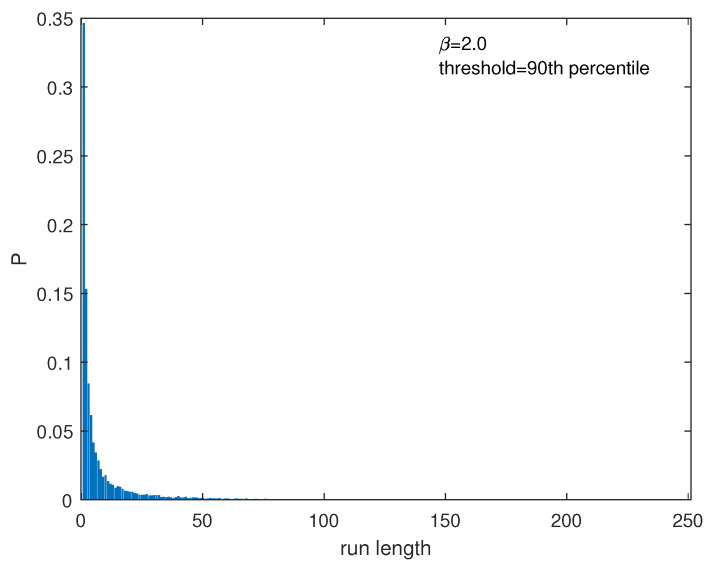
Probability distribution of run lengths of the extremes shown in Figure 2b.

We generated 10 time series, each comprising a length of *n* = 1,000,000 for every one of the six specified values of nonlinearity parameters, α (in the M1 model) and β (in the M2 model), and identified the extremes, forming temporal point processes for each series. This identification was carried out according to the methodology described in Section 3, employing four distinct percentile values (90th, 92.5th, 95th, and 97.5th). Subsequently, for each series of extremes, two distinct time series were derived—one representing inter-extreme times and the other the run lengths (resulting in a total of 960 series) (Figure 2 shows, as an example, the series of interevent times and that of the run lengths for one series generated with β=2.0 and for the threshold as the 90th percentile of the distribution of the series’ values). These individual time series were then analyzed by using the Fisher–Shannon Information Plane. The calculation of the $S_{n}\left[P\right]$ and F[P] involves the calculation of the discrete probability distribution *P*. Since both the interevent time and the run length are discrete variables, meaning they take only integer values, the probability density for each of them is discrete. Thus, for instance, if the run length *R* varies from $R_{min}$ to $R_{max},P\left(R\right)=\left\{p\left(R_{i}\right)=\frac{N_{R=R_{i}}}{N_{tot}},R_{i}=R_{min},...,R_{max}\right\}$. Figure 3 shows the probability distribution for the run lengths shown in Figure 2b. After calculating the probability distribution for each intervent time and run length series, we calculated $S_{n}\left[P\right]$ and F[P] by using Equations (9) and (10).

**Figure 4:**
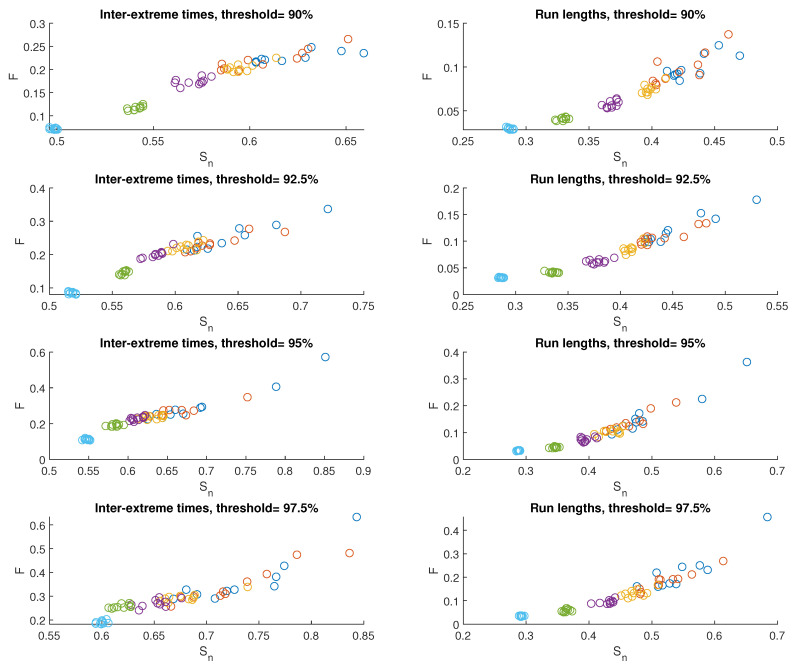
FSIP for both the series of inter-extreme time (**left**) and the run length series (**right**) derived from the data generated by model M1. The color of symbols refers to the following cases: $\alpha =\frac{1}{16}$ (blue), $\alpha =\frac{1}{8}$ (red), $\alpha =\frac{1}{4}$ (yellow), $\alpha =\frac{1}{2}$ (purple), α=1 (green), and α=2 (cyan)).

Figure 4 displays the results exhibited on the FSIP for both the series of inter-extreme time (presented on the left) and the run length series (shown on the right) derived from the data generated by M1. Different colors correspond to different values of the nonlinearity parameter α. There are 10 symbols of each color because 10 time series for each α have been produced.The widest variation in the results from these 10 series is observed for $S_{n}$, particularly for the case when $\alpha =\frac{1}{16}$. As α increases, this variation decreases.

**Figure 5:**
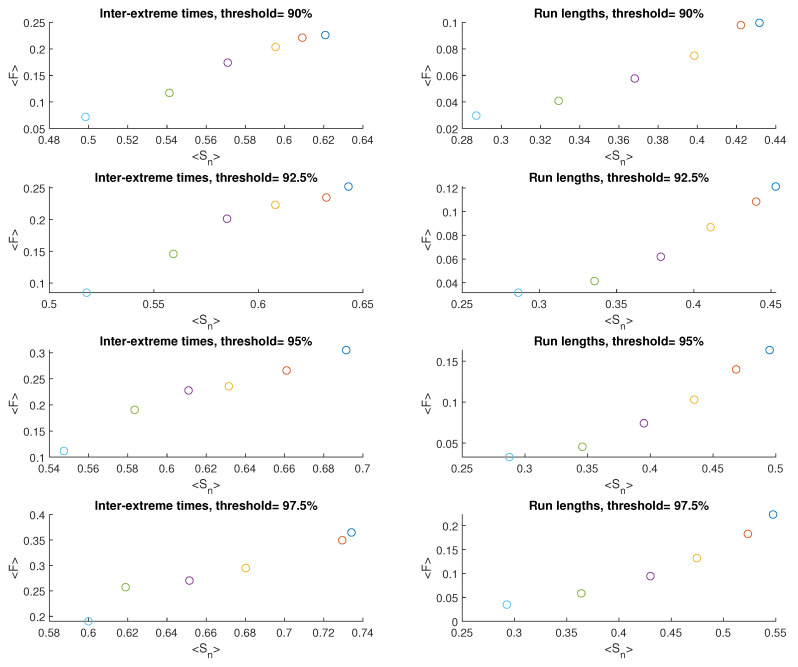
Averaged FSIP for both the series of inter-extreme time (**left**) and the run length series (**right**) derived from the data generated by model M1. The color of symbols refers to the following cases: $\alpha =\frac{1}{16}$ (blue), $\alpha =\frac{1}{8}$ (red), $\alpha =\frac{1}{4}$ (yellow), $\alpha =\frac{1}{2}$ (purple), α=1 (green), and α=2 (cyan).

In Figure 5, averaged values of $S_{n}$ and *F* are depicted. The average values of $S_{n}$ and *F* for the inter-extreme times decrease with an increase in the nonlinearity parameter α (ranging from $\frac{1}{16}$ to 2.0) for all analyzed threshold values. A similar trend is observed for the time series of run lengths.

**Figure 6:**
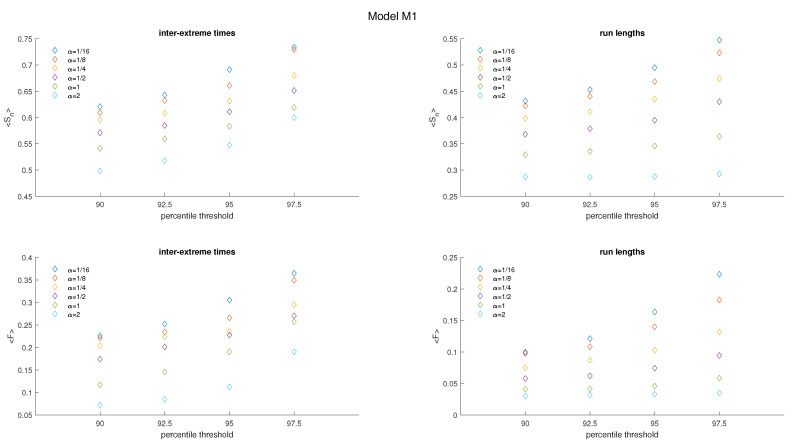
Average $S_{n}$ and *F* of inter-extreme times and run lengths versus the percentile threshold for the model M1.

An effect of the increasing threshold on $S_{n}$ and *F* is shown in Figure 6; we observe a growth of these quantities for inter-extreme times and run lengths.

**Figure 7:**
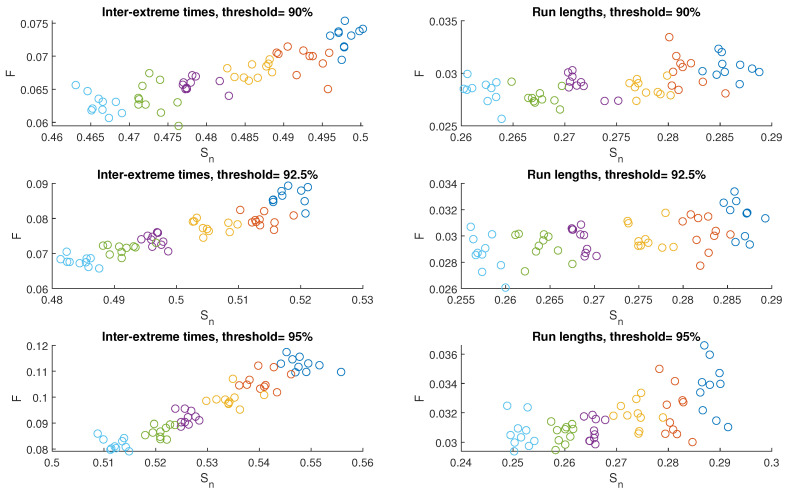

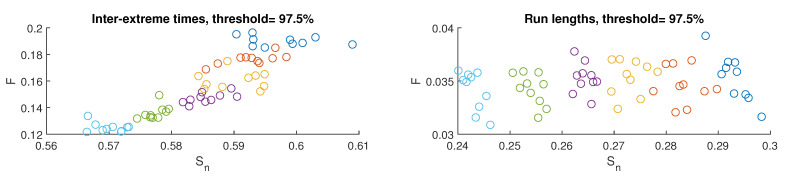
FSIP for both the series of inter-extreme time (**left**) and the run length series (**right**) derived from the data generated by model M2. The color of symbols refers to the following cases: β=2.0. (blue), β=2.2 (red), β=2.4 (yellow), β=2.6 (purple), β=2.8 (green), and β=3.0 (cyan).

**Figure 8:**
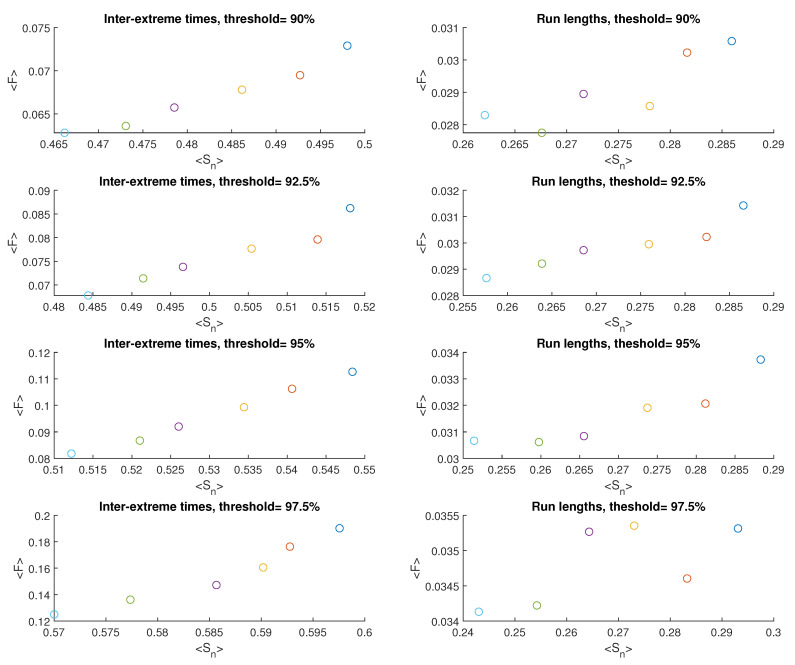
Averaged FSIP for both the series of inter-extreme time (**left**) and the run length series (**right**) derived from the data generated by model M2. The color of symbols refers to the following cases: β=2.0. (blue), β=2.2 (red), β=2.4 (yellow), β=2.6 (purple), β=2.8 (green), and β=3.0 (cyan).

Figure 7 and Figure 8 present graphs illustrating the outcomes obtained for model M2, similar to those in Figure 4 and Figure 5. The average value of $S_{n}$ and mean value of *F* for inter-extreme times decline with an increase in the nonlinearity parameter β (ranging from 2.0 to 3.0) across all analyzed threshold values (refer to Figure 8, plots on the left). However, for the time series of run lengths, the relationship holds only for $S_{n}$, whereas for *F*, a notable dispersion of results is observed (Figure 8, plots on the right).

**Figure 9:**
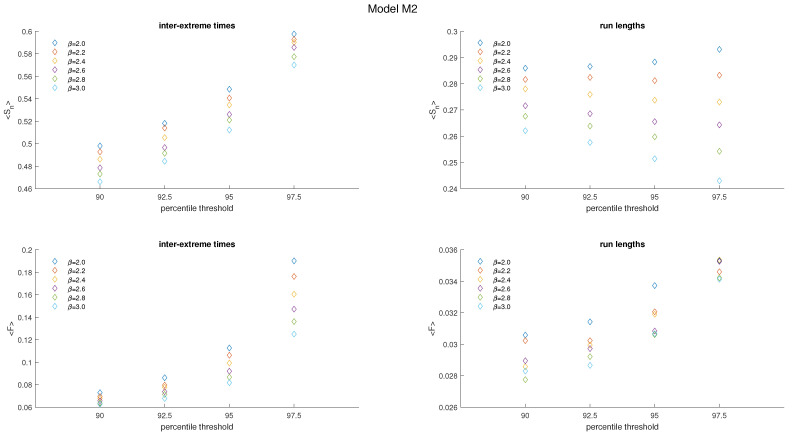
Average $S_{n}$ and *F* of inter-extreme times and run lengths versus the percentile threshold for the model M2.

When the threshold level increases, both $S_{n}$ and *F* for inter-extreme times increase, as shown in Figure 9, similar to M1. However, the behavior differs for run lengths in M2: $S_{n}$ increases for the case of β = 2.0, remains nearly constant for β = 2.2, but decreases for higher β values. On the other hand, *F* for run lengths maintains an overall increasing trend in most cases.

It is worth emphasizing that the range of changes in the values of these measures ($S_{n}$ and *F*) is considerably smaller in the M2 model compared to the M1 model.

## 6. Discussion

Natural complex phenomena often exhibit nonlinear behavior, necessitating appropriate nonlinear models to capture their underlying processes. Creating a precise deterministic model proves challenging due to our limited knowledge of degrees of freedom and internal mechanisms. Hence, stochastic models are commonly employed, approximating the effects of these processes via the introduction of random variables with specific probabilistic characteristics. This stochastic approach becomes invaluable when analyzing time series obtained from monitoring natural phenomena, particularly in studying extreme values due to their significant impact on safety, the economy, and health.

The objective of this study was to explore how different forms of nonlinearity within the stochastic model, specifically the Langevin equation, influence extreme behavior in generated time series. To facilitate our analysis, we introduced a single parameter to index the nonlinear variations in the drift and diffusion functions. We presented two models: in M1, the drift function varies along with the nonlinearity parameter, altering the shape of the marginal distribution function (shortening the tail of the distribution), while in M2, the form of the distribution function remains constant, and the two terms of the Langevin equation change accordingly. These models share a common aspect and smoothly transition from one to the other (for the largest assumed value of α in M1 and for the smallest assumed value of β in M2, both models become identical), unifying our analysis and interpretation.

To define and identify extremes, we employed run theory and percentile-based thresholds particularly suitable for hydrological and meteorological data analysis. We assessed extreme behavior on the Fisher–Shannon Information Plane, a widely used method for discriminating different measurement data. The analysis involved two types of extreme-related series: inter-extreme times and run lengths.

The Shannon Entropy, characterized by a global nature, measures the distribution spread and shows minimal sensitivity to abrupt changes in a localized distribution. In contrast, Fisher’s Information Measure exhibits a local property by focusing on the gradient content of the distribution. FIM reflects the irregularities of the density and then, it is a measure of systems disorder.

**Figure 10:**
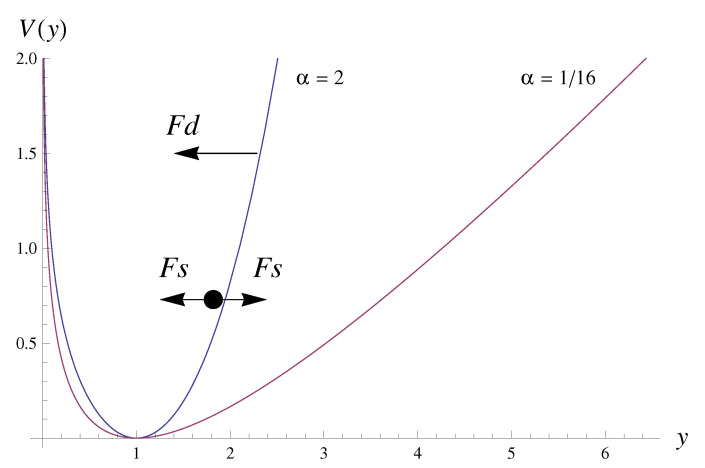
Graph of the potential V(y) for α= 2 (blue curve) and $\alpha =\frac{1}{16}$ (yellow curve). Arrows illustrate forces: $F_{s}$—stochastic forces directed to the right or to the left according to the sign of the diffusion term, $F_{d}$—repelling drift force.

To comprehend the behaviors of entropic and informational measures, $S_{n}$ and *F*, let us delve into the physical interpretation of the underlying Langevin equation. This equation encapsulates the diffusion of a particle within a potential niche (refer to Figure 10). The potential V(y) is represented by the drift function (dV(y)/dy=−a(y)), and the diffusion process, modeled by the white noise $\xi_{t}$, is modified by the diffusion function b(y). The competition or interaction of these two forces (drift force $F_{d}=a\left(y\right)$ and stochastic force $F_{s}={\left(b\left(y\right)/\Delta_{t}\right)}^{1/2}\xi_{t}$) describes the nonlinear dynamics of the process.

In Model M1, while the diffusion force remains constant across different α values, the right branch of the potential niche steepens with the increasing α (Figure 10). This restricts particle diffusion, limiting the *y* range and consequently shortening the tail of the marginal distribution (as seen in Figure 1). This constraint curtails chaotic movements, leading to a reduction in entropy. Our findings reveal that such characteristics are, to a certain extent, evident in the inter-extreme time and run length series of Model M1. The reduction in *F* with the increasing α is associated with decreased fluctuations, thereby reducing uncertainty in distribution function estimation of inter-extreme time and run length from time series. Although these distributions become narrower (resulting in an increased *F*), their ‘smoothness’ prevails, leading to a decrease in *F*, which dominates.

In the case of Model M2, the marginal distribution of the background series remains constant across different β values; therefore, both the informational quantities for these series do not change with the parameter. However, the nonlinear dynamics, influenced by varying forms of drift and diffusion forces (see Equation (4)), significantly impact extreme behaviors, as indicated rather well by $S_{n}$. While the distributions of inter-extreme time and run length series become narrower (yet ’smoother’) with the increasing β, the decrease in *F* for the run lengths is no longer dominant, but adopts a compensatory nature.

## 7. Conclusions

Our findings demonstrate a distinct impact of varying forms of nonlinearity, represented by parameters α and β, on the behavior of extremes. With an increase in α, we observed a shift of states towards lower values of *F* and $S_{n}$ on the FSIP for inter-extreme times, which continued at a slower pace with an increase in β in the M2 model. A similar trend was noticeable for run lengths in M1, while in M2, *F* and $S_{n}$ values related to run lengths exhibited fluctuations that disrupted the trend. As the threshold level increases, these trends are sustained, with both *F* and $S_{n}$ increasing. The observed shifts on the FSIP can be partially explained based on the physical interpretation of the Langevin equation.

A crucial outcome of this study is that even if the nonlinearity in the drift and diffusion terms of the Langevin equation does not alter the marginal distribution function, it still influences the behavior of extreme values. Enhancing our understanding of how the nonlinear form of the Langevin equation impacts the behavior of extremes in generated time series will help in constructing more suitable stochastic models for underlying processes.

## Data Availability

The data that support the findings of this study are available from the corresponding author upon reasonable request.

## Supplementary Materials

The following supporting information can be downloaded at: https://www.mdpi.com/article/10.3390/e25121650/s1.
