# Supplementary material for: Fisher–Shannon Investigation of the Effect of Nonlinearity of Discrete Langevin Model on Behavior of Extremes in Generated Time Series
Source: Entropy (Basel). 2023 Dec 12;25(12):1650. doi: 10.3390/e25121650 (PMC10742732; doi:10.3390/e25121650)
Supplement: Supplementary file 1 [file entropy-25-01650-s001.zip › entropy-2737974-supplementary.pdf]

Article

Fisher-Shannon investigation of the effect of nonlinearity of discrete Langevin model on behavior of extremes in generated time series. Supplementary information

Luciano Telesca [1,*] and Zbigniew Czechowski [2]

[1] Institute of Methodologies for Environmental Analysis, National Research Council, 85050 Tito, Italy; luciano.telesca@imaa.cnr.it
[2] Institute of Geophysics, Polish Academy of Sciences, 01-452 Warsaw, Poland; zczech@igf.edu.pl
* Correspondence: luciano.telesca@imaa.cnr.it (L.T.)

Considering discrete probability distributions ($P = \{p_j : j = 1, \cdots, M\}$), the FIM is defined as:

$$F[P] = F_0 \sum_{i=1}^{M-1} \left[(p_{i+1})^{\frac{1}{2}} - (p_i)^{\frac{1}{2}} \right]^2 \tag{1}$$

The normalization constant is defined as:

$$F_0 = \begin{cases} 1 & \text{if} \quad p_{i^*} = 1 \quad \text{for} \quad i^* = 1 \quad \text{or} \quad i^* = M \quad \text{and} \quad p_i = 0 \quad \forall \quad i \neq i^* \\ \frac{1}{2} & \text{otherwise} \end{cases}$$

The normalization constant ensures that the maximum FIM is set to 1. In fact:

$$\begin{aligned} \sum_{i=1}^{M-1} \left[(p_{i+1})^{\frac{1}{2}} - (p_i)^{\frac{1}{2}} \right]^2 = & \\ & (\sqrt{p_1})^2 + 2(\sqrt{p_2})^2 + \ldots + 2(\sqrt{p_{M-1}}) + (\sqrt{p_M})^2 + \\ & - 2(\sqrt{p_1}\sqrt{p_2} + \sqrt{p_2}\sqrt{p_3} + \cdots + \sqrt{p_{M-1}}\sqrt{p_M}) = \\ & = p_1 + 2p_2 + \ldots + 2p_{M-1} + p_M + \\ & - 2(\sqrt{p_1}\sqrt{p_2} + \sqrt{p_2}\sqrt{p_3} + \cdots + \sqrt{p_{M-1}}\sqrt{p_M}) = \\ & 1 + (p_2 + p_3 + \ldots + p_{M-1}) + \\ & - 2(\sqrt{p_1}\sqrt{p_2} + \sqrt{p_2}\sqrt{p_3} + \cdots + \sqrt{p_{M-1}}\sqrt{p_M}) = \\ & 1 + 1 - (p_1 + p_M) - 2(\sqrt{p_1}\sqrt{p_2} + \cdots + \sqrt{p_{M-1}}\sqrt{p_M}) \leq 2 \end{aligned} \tag{2}$$

Thus

$$F[P] = F_0 \sum_{i=1}^{M-1} \left[(p_{i+1})^{\frac{1}{2}} - (p_i)^{\frac{1}{2}} \right]^2 \leq 1 \tag{3}$$
